# Structure–function study of a novel inhibitor of the casein kinase 1 family in *Arabidopsis thaliana*

**DOI:** 10.1002/pld3.172

**Published:** 2019-09-17

**Authors:** Ami N. Saito, Hiromi Matsuo, Keiko Kuwata, Azusa Ono, Toshinori Kinoshita, Junichiro Yamaguchi, Norihito Nakamichi

**Affiliations:** ^1^ Department of Applied Chemistry Waseda University Shinjuku, Tokyo Japan; ^2^ Institute of Transformative Bio‐molecules (WPI‐ITbM) Nagoya University Chikusa, Nagoya Japan; ^3^ Division of Biological Science Graduate School of Science Nagoya University Chikusa, Nagoya Japan

**Keywords:** Arabidopsis, casein kinase 1, circadian clock, inhibitor, structure–activity relationship

## Abstract

Casein kinase 1 (CK1) is an evolutionarily conserved protein kinase family among eukaryotes. Studies in non‐plants have shown CK1‐dependent divergent biological processes, but the collective knowledge regarding the biological roles of plant CK1 lags far behind other members of the Eukarya. One reason for this is that plants have many more genes encoding CK1 than do animals. To accelerate our understanding of the plant CK1 family, a strong CK1 inhibitor that efficiently inhibits multiple members of the CK1 protein family in vivo (*i.e.,* in planta) is required. Here, we report a novel, specific, and effective CK1 inhibitor in Arabidopsis. Using circadian period‐lengthening activity as an estimation of the CK1 inhibitor effect in vivo, we performed a structure–activity relationship study of analogues of the CK1 inhibitor PHA767491 (1,5,6,7‐tetrahydro‐2‐(4‐pyridinyl)‐4H‐pyrrolo[3,2‐c]pyridin‐4‐one hydrochloride). A propargyl group at the pyrrole nitrogen atom (AMI‐212) or a bromine atom at the pyrrole C3 position (AMI‐23) had stronger CK1 inhibitory activity than PHA767491. A hybrid molecule of AMI‐212 and AMI‐23 (AMI‐331) was about 100‐fold more inhibitory than the parent molecule PHA767491. Affinity proteomics using an AMI‐331 probe showed that the targets of AMI‐331 inhibition are mostly CK1 kinases. As such, AMI‐331 is a potent and selective CK1 inhibitor that shows promise in the research of CK1 in plants.

## 1 INTRODUCTION

Casein kinase 1 (CK1) is a serine–threonine protein kinase that is evolutionarily conserved among eukaryotes. CK1 plays a number of key roles in biological processes such as DNA damage signal transduction and repair, cytokinesis, cell cycle progression and apoptosis, immune responses, vesicular trafficking, signaling pathways for development, and regulation of circadian rhythms (Gorl et al., 2001; Gross & Anderson, 1998; Knippschild et al., 2014).

In the model plant *Arabidopsis thaliana* (Arabidopsis), CK1 family kinases regulate stomatal closure (Zhao et al., 2016), blue‐light signaling (Tan, Dai, Liu, & Xue, 2013), cortical microtubules (Ben‐Nissan et al., 2008), and ethylene production (Tan & Xue, 2014). A subset of CK1 proteins in Arabidopsis known as CASEIN KINASE 1 LIKE (CKL) are known to phosphorylate substrate proteins, and phosphorylation by CKLs triggers one of two distinct effects: degradation of substrates through the ubiquitination pathway, or modification of substrate activity. Phosphorylation of CRYPTOCHROME (CRY), a protein involved in the blue‐light signaling pathway as mediated by CKL3 and CKL4, is related to CRY degradation (Tan et al., 2013). CKL8 is involved in controlling the degradation of 1‐AMINOCYCLOPROPANE‐1‐CARBOXYLIC ACID SYNTHASE 5 (ACS5) by phosphorylation during ethylene synthesis (Tan & Xue, 2014). CKL2 regulates F‐actin disassembly of ACTIN‐DEPOLYMERIZING FACTOR 4 (ADF4) by phosphorylation (Zhao et al., 2016). CKL6 controls microtubule dynamics by phosphorylating tubulin (Ben‐Nissan et al., 2008). Rice Hybrid breakdown 2 (Hdb2) belongs to the CK1 family and is known to be involved in regulating reproductive isolation or hybrid breakdown (Yamamoto et al., 2010), root development, and hormone sensitivity (Liu, Xu, Luo, & Xue, 2003), although the specific substrates of rice CK1 have not been identified.

MUT9‐LIKE KINASES (MLKs), also known as PHOTOREGULATORY PROTEIN KINASE (PPK) proteins, are the plant kinases that most resemble the CKL family (Huang et al., 2015; Liu et al., 2017; Ni et al., 2017; Wang et al., 2015). Arabidopsis MLKs/PPKs are involved in both light signaling and regulation of the circadian clock. MLKs/PPKs interact with Evening Complex components [LUXARRHYTHMO (LUX), EARLY FLOWERING 3 (ELF3), and ELF4] of the circadian clock (Huang et al., 2015), and with CRY and PHYTOCHROME INTERACTING FACTOR (PIF) proteins in blue‐ and red‐light signaling (Liu et al., 2017; Ni et al., 2017). Rice Heading date 16 (Hd16) had been proposed as a regulator of flowering time and was at one time considered to be a rice CK1 homologue (Hori et al., 2013). However, phylogenic analysis indicates that Hd16 is more properly placed in the MLK/PPK family (Hori et al., 2013). Hd16 phosphorylates Hd2/pseudo‐response regulator 37 (OsPRR37) and grain number, plant height and heading date 7 (Ghd7) in vitro (Hori et al., 2013; Kwon, Koo, Kim, Yoo, & Paek, 2015), and controls photoperiodic flowering time in rice, but it does not strongly affect circadian clock parameters (Hori et al., 2013).

Extensive genetic redundancy among multiple members of the CKL subfamily (e.g., the 13 CKLs in Arabidopsis) may make further delineation of the biological processes regulated by the CK1 family challenging because of the difficulty in eliminating kinase function by knocking out or knocking down gene expression of one or combinations of CK1 family genes. To meet this challenge, small molecule inhibitors of CK1 can be employed to determine whether or not CK1 enzymes are involved in a given biological process (Uehara et al., 2019). The small molecule IC261 has mostly been used for this purpose, and more recent studies used PF‐670462, which is a more potent and specific inhibitor of plant CK1 enzymes (Mizoi et al., 2019; Uehara et al., 2019). Chemical screening combined with target identification of the target molecule indicated that PHA767491, a mammalian CDC7 (Cell division control protein 7) inhibitor, also targets plant CK1 (Uehara et al., 2019). A combination of PF‐670462 and PHA767491 demonstrated that CK1 is involved in Arabidopsis circadian clock regulation (Uehara et al., 2019). PHA767491 does not bind to MLKs/PPKs (Uehara et al., 2019), though the amino acid sequence similarity between kinase domains of MKLs/PPKs and those of CK1 is about 40% (Liu et al., 2017). Therefore, utilization of potent CK1 inhibitors can be used to reveal the biological processes controlled by CK1. However, the concentration of these molecules required to modulate biological processes, or to produce measurable phenotypes, was around 100 µM. This high concentration may have harmful and confounding physiological effects. PHA767491 binds not only to CK1 family proteins, but also to other kinases, such as SHAGGY‐LIKE KINASEs (ATSKs, also called GSK3s), CALCIUM‐DEPENDENT PROTEIN KINASEs (CPKs), MITOGEN‐ACTIVATED PROTEIN KINASE (MPKs), and other kinases, therefore showing relatively low specificity for CK1 (Uehara et al., 2019). Reducing the effective concentration and increasing the specificity of CK1 inhibitors would enhance their utility. An inhibitor with higher specificity that could be used at a very low concentration would also reduce the possibility that PHA767491 modulates the clock via CK1‐independent mechanisms.

Through structure–activity relationship studies of modifications of the plant CK1 inhibitor PHA767491, here we describe a small molecule with the strongest known CK1 inhibitory activity in vivo. This molecule, named AMI‐331, significantly lengthens the Arabidopsis circadian clock period at concentrations below 1 µM with high specificity for CK1 family kinases.

## 2 MATERIALS AND METHODS

### 2.1 Plant materials and growth conditions

Arabidopsis Columbia‐0 (Col‐0) accession plants were used as wild‐type. *CCA1:LUC* (Nakamichi, Kita, Ito, Yamashino, & Mizuno, 2005) and *35Spro:PRR5‐FLAG*, *35Spro:PRR5‐VP*, and *35Spro:TOC1‐VP* plants were described previously (Nakamichi et al., 2016). Plants were grown on full strength of Murashige‐Skoog (MS) containing 2% sucrose and 0.3% gellan gum under 12‐hr white light (70 µmol/s m^−2^)/12‐hr dark conditions.

### 2.2 In vitro phosphorylation assays of Arabidopsis CKL4

In vitro phosphorylation assays using recombinant CKL4 were performed as described previously (Uehara et al., 2019), with synthetic small molecules. IC261 and PF‐670462 were purchased (Sigma‐Aldrich catalog numbers I0658 and SML0795, respectively). PHA767491 was synthesized as previously described (Uehara et al., 2019). All chemical compounds were dissolved in dimethyl sulfoxide (DMSO) as 10 mM stock solutions. Stock solutions were diluted with DMSO to the working concentration and added to assays in kinase reaction buffer (Uehara et al., 2019).

### 2.3 Bioluminescence‐based circadian rhythm

Bioluminescence‐based circadian rhythms of *CCA1:LUC* plants treated with small molecules were analyzed by auto‐luminescence detection (Churitsu CL96) as described previously (Uehara et al., 2019). Period length was automatically calculated as previously described (Kamioka et al., 2016).

### 2.4 Synthesis of PHA767491 analogues (AMI molecules)

Synthesis of PHA767491 analogues is described in Supporting information. AMI‐331 for basic plant research is now commercially available (Tokyo Chemical Industry, product No. A3352).

### 2.5 Western blotting

Four‐day‐old seedlings grown under 12‐hr light/12‐hr dark (LD) conditions were transferred into a 96‐well plate with a dropper. Seedlings were treated with 20 µl of MS liquid containing 2% sucrose and AMI‐331 at 2, 10, or 50 µM with a final concentration of 5% (v/v) DMSO. As a control experiment, MS containing 2% sucrose and 5% DMSO was used to treat the seedlings. Seedlings were kept under constant light (L) or constant dark (D) for 24 hr, harvested, and kept frozen until proteins were extracted. Frozen samples were crushed with zirconia beads (Tomy ZB‐50) in a Tissue Lyser II (Qiagen). Detection of PRR5‐ and TOC1‐fusion proteins was performed using a 10%–20% gradient acrylamide gel (198‐15041, Wako) as previously described (Nakamichi et al., 2012). Anti‐FLAG antibody (F3165, Sigma) and anti‐VP antibody (ab4808, Abcam) were used to detect FLAG‐fusion and VP‐fusion proteins, respectively.

### 2.6 Screening of proteins bound to AMI‐329 beads

Screening for proteins bound to AMI‐329 beads was done by a method similar to what has previously been described (Uehara et al., 2019). Briefly, two‐week‐old seedlings grown under LD conditions were harvested at time points ZT2, ZT6, ZT9, and ZT17, and stored at −80°C. Proteins from the frozen plant samples were incubated with 0, 5, or 50 µM of AMI‐331 at 4°C for 30 min with rotary mixing. Two technical replicates were used for each experiment. PBS‐washed AMI‐329 beads were added to the protein samples and gently rotated at 4°C for 1 hr. AMI‐329 bead resins were washed with bead buffer (Uehara et al., 2019) six times. The washed resins were suspended in SDS sample buffer and boiled for 8 min. Protein samples were in‐gel‐digested as previously described (Uehara et al., 2019). Peptides were analyzed with a Q Exactive hybrid quadrupole‐orbitrap mass spectrometer (Thermo Fisher Scientific), as described previously (Uehara et al., 2019). MS/MS spectra were interpreted, and peak lists were generated using Proteome Discoverer 2.0.0.802 (Thermo Fisher Scientific). Searches were performed using SEQUEST (Thermo Fisher Scientific) against the *Arabidopsis thaliana* (TAIR TaxID = 3,702) peptide sequence database.

Once the two technical replicates were shown to have similar results, spectra data of the technical replicates were merged. Proteins whose digested peptides’ spectra were over “2” in the 0 µM AMI‐331 sample were selected to ensure data integrity. We then selected proteins whose relative spectra (spectra in 0 µM/summed spectra in 5 and 50 µM) were over “10,” as AMI‐331‐bound proteins (Figure 5c). To provide overview spectra for potential PHA767491‐target proteins (Uehara et al., 2019), spectra of these proteins were obtained from AMI‐329 bead‐bound samples, and relative spectra against 0 µM of AMI‐331 samples were shown (Figure 6).

### 2.7 Gene expression analysis

Arabidopsis Col‐0 seedlings that had been grown under constant light conditions for 6 days were immersed in 10 or 50 µM of the GSK‐3, SHAGGY‐related, and BIN2 kinase inhibitor Bikinin (SML0094, Sigma) (De Rybel et al., 2009), 1, 5, or 20 µM of AMI‐331, or DMSO as control for 6 hr. Three biological replicates were used for each assay. Harvested seedlings were frozen in liquid nitrogen and crushed with zirconia beads in a Tissue Lyser II, and RNA was isolated with Illustra RNAspin (25‐0500‐72, GE Healthcare). RT‐qPCR was performed as described previously (Nakamichi et al., 2010) using an Eco Real‐Time PCR System (Illumina). Gene expression was normalized against *IPP2*, and maximal values were set to 1. Primers for detecting *IPP2*, *PRR7*, *GR60ox2*, and *CPD* were described previously (De Rybel et al., 2009; Kamioka et al., 2016).

### 2.8 Accession numbers

Sequence data for the genes described in this article are found in the Arabidopsis Information Resource under following numbers: *CCA1* (At2g46830), *CPD* (At5g05690), *IPP2* (AT3G02780) *GR60ox2* (At3g30180), and *PRR7* (At5g02810).

## 3 RESULTS

### 3.1 Three CK1 inhibitors lengthen the circadian period

**Figure 1:**
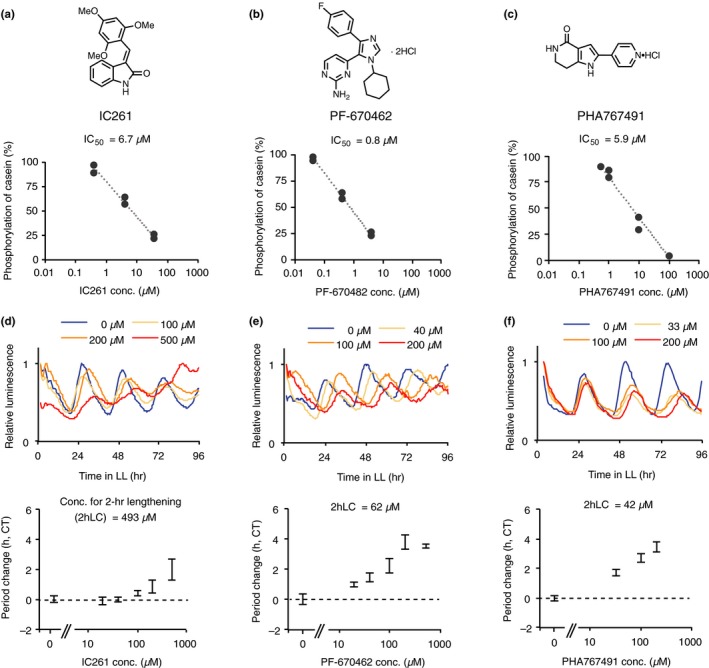
Activities of IC261, PF‐670462, and PHA767491. Chemical structures of IC261 (a), PF‐670462 (b), and PHA767491 (c). In vitro CKL4 kinase activity in assays with IC261 (a), PF‐670462 (b), or PHA767491 (c). Activity of the circadian luciferase reporter *CCA1:LUC* with IC261 (d), PF‐670462 (e), or PHA767491 (f). (d–f), Representative traces of a single replicate (upper) and increasing of period length relative to control experiments (lower). Error bars indicate *SEM* (*n* = 3–12) in lower panels. 2hLC indicates the concentration required for 2‐hr period lengthening. Similar data were obtained from other trials (d–f)

The activities of three known CK1 inhibitors were measured in vitro by a previously reported method (Uehara et al., 2019). Recombinant CKL4 kinase, casein, ^32^P‐ATP, and different concentrations of the inhibitors were combined in a reaction buffer and kept at 37°C for 2 hr. Resulting samples were separated on a polyacrylamide gel by electrophoresis, and ^32^P phosphorylation of casein was measured as an indicator of CKL4 kinase activity. IC_50_ (half‐maximal inhibitory concentration) was determined by calculating the results from at least two independent experiments. IC_50_ of IC261, PF‐670462, and PHA767491 were 6.7, 0.8, and 5.9 µM, respectively (Figure 1a–c). The stronger in vitro CK1 inhibitory activity shown by PF‐670462 relative to PHA767491 was consistent with previous reports (Uehara et al., 2019).

To estimate CK1 inhibitor activity in vivo, we choose to measure circadian period‐lengthening activity in Arabidopsis seedlings. The assay was made more efficient by monitoring the circadian period using a clock reporter plant line (*CCA1:LUC*) and an automated luminometer. These tools can be used to measure the circadian period with high confidence, and estimates of in vivo CK1 inhibitor activity are quantitative (Uehara et al., 2019). Four‐day‐old *CCA1:LUC* seedlings grown under 12‐hr light/12‐hr dark conditions were treated with IC261, PF‐670462, and PHA767491 at different concentrations, and seedling luminescence was monitored under constant light conditions. Dimethyl sulfoxide (DMSO), the solvent used for all of the CK1 inhibitors, was used as a control. IC261 lengthened the circadian period of *CCA1:LUC* seedlings in a dose‐dependent manner (Figure 1d), with a 2‐hr period lengthening at 500 µM. PF‐670462 and PHA767491 treatment concentrations also correlated with period lengthening (Figure 1e and f). The concentrations required for 2‐hr lengthening effects were 90 µM for PF‐670472 and 47 µM for PHA767491, which was consistent with previous work (Uehara et al., 2019). Thus, PHA767491 was the strongest in vivo CK1 inhibitor among the three inhibitors tested. This contrasts with in vitro CK1 inhibitory activity, where PHA767491 was less effective than PF‐670462.

### 3.2 Pyrrole ring derivatives of PHA767491 have strong period‐lengthening activities

**Figure 2:**
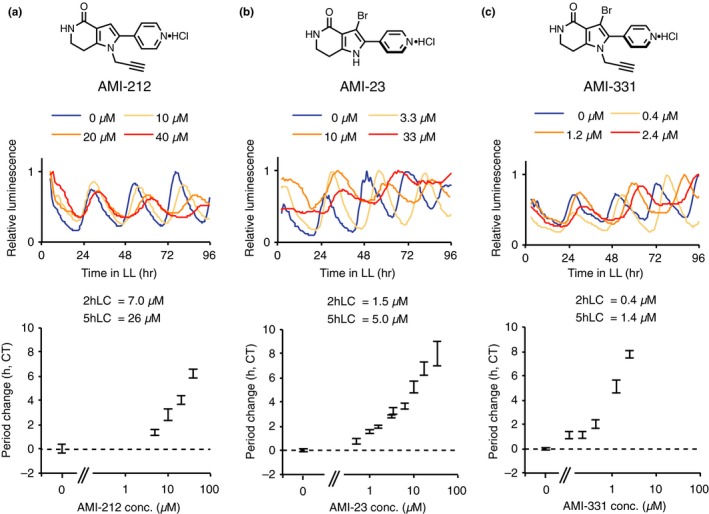
Period‐lengthening activity of AMI‐212, AMI‐23, and AMI‐311. Structures of AMI‐212 (a), AMI‐23 (b), and AMI‐331 (c) (upper). *CCA:LUC* activity with AMI‐212 (a), AMI‐23 (b), or AMI‐331 (c) (middle and lower). Middle panels show representative traces of a single replicate. Lower panels show increasing period lengths relative to controls (*n* = 8–22, with *SEM*). 2hLC and 5hLC indicate the concentrations required for 2‐hr and 5‐hr period lengthening, respectively. The data were combined from at least two separate trials

We sought to create a more potent CK1 inhibitor by modifying the structure of PHA767491, since the in vitro CK1 inhibitor activity of PHA767471 was not as high as PF‐670472. We applied previously published synthetic methods to make further derivatives of PHA767491 (Uehara et al., 2019), focusing on modification of the pyrrole at the N‐position, because two previously synthesized derivatives modified at this position retained period‐lengthening activity (Uehara et al., 2019). The circadian period lengths of *CCA1:LUC* seedlings treated with newly synthesized PHA767491 derivatives were measured as described above (Figure S1). Derivatives with either a large substituent group at the pyrrole N‐position retained weak period‐lengthening activity compared to the PHA767491 parent compound (e.g., AMI‐118, AMI‐212). On the other hand, derivatives with an ethyl group at the pyrrole N‐position possessed strong activity (AMI‐126). Derivatization of AMI‐126 with small alkyl groups also had enhanced activity. Among these derivatives, AMI‐212 (Figure 2a) had stronger period‐lengthening activity than AMI‐126. Further analysis showed that AMI‐212 concentrations around 7.0 µM lengthened the circadian period by about 2 hr, and 26 µM lengthened the period by about 5 hr. AMI‐212 therefore had approximately fivefold higher inhibitory activity than PHA767491 (Figure 2a).

Strong activities were also associated with three derivatives with bromo‐, chloro‐ or methyl‐groups at the pyrrole C3 position (AMI‐23, 24, and 217, Figure S1). These substituents cause conformational twisting between the pyrrole and 4‐pyridyl rings. Treatment with 1.5 µM AMI‐23 lengthened the circadian period by 2 hr and 5.0 µM lengthened it by 5 hr (Figure 2b).

A hybrid molecule of AMI‐212 and AMI‐23 was generated to combine the features of these two period‐lengthening compounds, resulting in AMI‐331. AMI‐331 concentrations of 0.4 and 1.4 µM lengthened the period by about 2 and 5 hr, respectively (Figure 2c), or at about one‐hundredth the concentration of PHA767491.

### 3.3 Over‐accumulation of two clock‐associated transcription factors with AMI‐331 treatment

**Figure 3:**
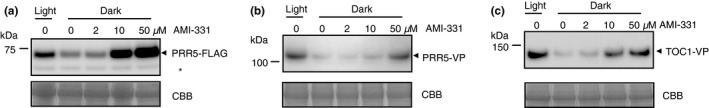
PRR5 and TOC1 protein accumulation in plants treated with AMI‐331. Relative protein accumulation of PRR5‐FLAG (a), PRR5‐VP (b), and TOC1‐VP (c) in transgenic plants treated with AMI‐331 (upper). CBB‐stained gel was used to indicate that similar amounts of proteins were used (lower). Similar results were obtained in another trial (Figure S2)

It had previously been shown that CKL4 phosphorylates PSEUDO‐RESPONSE REGULATOR 5 (PRR5), a clock‐associated transcription factor in vitro, and that treatment with the CK1 inhibitor PHA767491 results in over‐accumulation of PRR5 in vivo (Uehara et al., 2019). As a further test of whether the period‐lengthening activity of AMI‐331 is related to the inhibition of CK1 in vivo, we measured the amounts of PRR5 in plants treated with AMI‐331. Seedlings expressing fusion protein PRR5‐FLAG under control of the cauliflower mosaic virus 35S promoter (*35Spro:PRR5‐FLAG*) were grown under constant light conditions for 4 days, treated with AMI‐331, and kept constantly in the dark for one day. In control experiments (*i.e.,* without AMI‐331), PRR5‐FLAG protein amounts were lower under dark compared to light conditions, due to ZTL‐dependent degradation, as described in previously (Fujiwara et al., 2008; Kiba, Henriques, Sakakibara, & Chua, 2007). AMI‐331 treatment resulted in over‐accumulation of PRR5‐FLAG under dark conditions (Figures 3a, S2). To determine if AMI‐331 treatment also results in accumulation of PRR5‐VP, we measured this protein in *35Spro:PRR5‐VP*, which has the reverse phenotype of *35Spro:PRR5‐FLAG*. Treatment with AMI‐331 resulted in accumulation of PRR5‐VP (Figures 3b, S2) in a dose‐dependent manner. Since PHA767491 treatment also attenuates degradation of TIMING OF CAB EXPRESSION 1 (TOC1, also called as PRR1) under dark conditions (Uehara et al., 2019), we tested whether AMI‐331 affects TOC1 protein accumulation in vivo. TOC1 amounts were higher in AMI‐331‐treated plants (Figures 3c, S2). The effective concentrations of AMI‐331 for PRR5 or TOC1 accumulation (10–50 µM) were far less than for PHA767491 (250–500 µM) (Uehara et al., 2019), indicating that AMI‐331 has higher kinase inhibition activity than PHA767491 in vivo.

### 3.4 AMI‐331 has strong CK1 inhibitory activity in vitro

**Figure 4:**
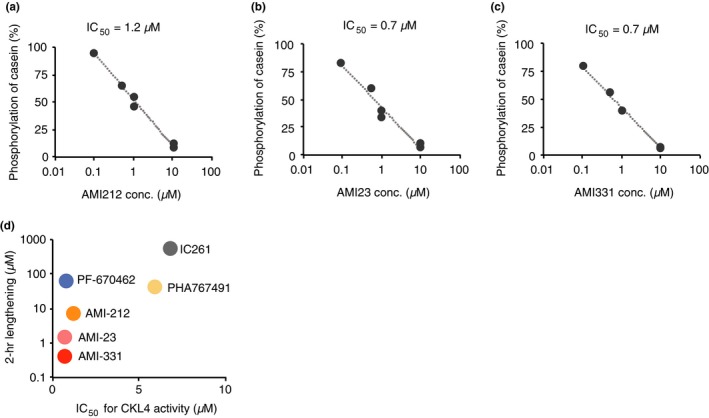
In vitro CKL4 kinase activity with three AMI‐derivative molecules. In vitro CKL4 kinase activity with AMI‐212 (a), AMI‐23 (b), or AMI‐331 (c). (d) Comparisons of in vitro CK4 kinase activity (*x*‐axis) and in vivo period‐lengthening activity (*y*‐axis) with the CK1 inhibitors

The IC_50_ value for CKL4 kinase activity in vitro with AMI‐212 treatment was 1.2 µM, and AMI‐23, and AMI‐331 had IC_50_ values 0.7 µM (Figure 4). The IC_50_ for AMI‐331 on CKL1 activity was 1.4 µM (Figure S3). The IC_50_ for AMI‐331 was about five times lower than for PHA767491 (Figure 1). These results suggest that the strong in vitro CK1 inhibitory activity of AMI‐331 is responsible for the correlated CK1 inhibitory activity in vivo, based on period‐lengthening activity and accumulation of PRR5 and TOC1. However, the extensive period‐lengthening activity that results from AMI‐331 treatment compared to PHA767491 at one‐hundredth the concentration, as well as PRR5 accumulation activity at one‐fiftieth the concentration, suggests that there are pharmacological properties of AMI‐331 which have not been accounted for that contribute to its strong in vivo period‐lengthening activity.

### 3.5 Target identification of AMI‐311

**Figure 5:**
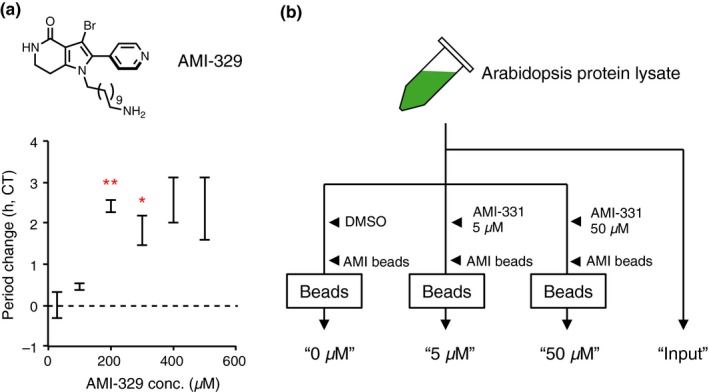
Procedure for target identification of AMI‐331. (a) Structure and period‐lengthening activity of AMI‐329. Asterisks indicate significant period changes compared to control samples (Bonferroni correction *p* < .05 (*) and 0.01 (**), respectively). (b) Procedure for identification of AMI‐331 targets, which should be enriched in “0 µM” compared to “5 µM,” “50 µM,” since free AMI‐331 and AMI‐329 beads competitively bind to AMI‐331 targets

The strong in vitro and in vivo CK1 clock‐related AMI‐331 inhibitory activity demonstrated in these experiments suggested that clock modulation is by direct inhibition of CK1. However, another possible mechanism for the strong AMI‐331 biological activity in vivo is that it targets proteins other than the CK1 family for period lengthening. To test this possibility, we synthesized molecular probes to screen direct target proteins of AMI‐331. Analogues substituted with an alkyl group at the nitrogen atom of the pyrrole ring of PHA767491 retain period‐lengthening activity (Figure 2, Figure S1, Uehara et al., 2019); therefore, an alkyl linker was attached at the pyrrole N of AMI‐331, generating AMI‐329 (Figure 5a). AMI‐329 retained weak but significant period‐lengthening activity (Figure 5a). AMI‐329 was then covalently bound to agarose beads and mixed with Arabidopsis seedling protein lysates, with or without AMI‐331 (0, 5 or 50 µM) as competitor (Figure 5b). The resulting peptide spectra showing “1” in the MS analysis may be due to false‐positive or background noise. Therefore, we used only proteins with digested peptide spectra that were > “2” in the no‐competitor (0 µM) samples to ensure data integrity (Table 1). We further selected proteins from this group with relative spectra (spectra in 0 µM/summed spectra in 5 and 50 µM) were > “10,” as AMI‐331‐bound proteins. These criteria resulted in a set of 23 proteins that included all members of the CK1 family and an additional ten non‐CK1 proteins (Table 1). Spectra of these proteins ranged from 0% to 7% in the 5 µM AMI‐331 sample, compared to the control sample without competitor (0 µM). The spectra of these proteins were absent from the sample containing 50 µM AMI‐331 as competitor. A few other proteins were enriched in the 0 µM sample compared to the 5 and 50 µM samples, but at amounts less than those found in the CKL family (Table 1). It is noteworthy that kinase AT4G08800 is very similar to the CKL family, but the ATP‐binding pocket of mammalian CK1 as determined by its crystal structure (Shinohara et al., 2017) was absent from AT4G08800, as shown in an Araport 11 model (https://www.araport.org). Therefore, we were not able to conclude that AT4G08800 is a CKL protein, per se, in this study. Spectra of a reductase C (AT2G41680) and an unknown protein (AT5G42765) in the input fraction were higher than in the 0 µM sample. Collectively, this analysis suggests that AMI‐331 is most specific for the CKL family, but that it also has some binding affinities to HYDRA1 (HYD1), LUPEOL SYNTHASE 1 (LUP1), YEAST YAK1‐RELATED GENE 1 (YAK1), NRPB3, RNA‐binding protein AT3g15010, two possible kinases (AT4G08800 and AT4G34500), and membrane protein AT5G40670.

**Table 1:** Proteins bound by AMI‐329 beads

		Spectra (PSMs)^a^			
		AMI‐329 beads bound			Input
		Competitor (AMI‐331) conc.			
Name	AGI code	0	5	50 µM	
CKL1	AT4G26100	35	2	0	1
CKL2	AT1G72710	31	1	0	0
CKL3	AT4G28880	76	1	0	0
CKL4	AT4G28860	45	1	0	0
CKL5	AT2G19470	32	1	0	0
CKL6	AT4G28540	26	1	0	0
CKL7	AT5G44100	31	1	0	0
CKL8	AT5G43320	8	0	0	0
CKL9	AT1G03930	31	1	0	0
CKL10	AT3G23340	25	1	0	0
CKL11	AT4G14340	25	1	0	0
CKL12	AT5G57015	30	2	0	1
CKL13	AT1G04440	8	0	0	0
Kinase	AT4G08800	6	0	0	0
HYD1	AT1G20050	2	0	0	0
Kinase	AT4G34500	3	0	0	0
LUP1	AT1G78970	2	0	0	0
Membrane protein	AT5G40670	3	0	0	0
NRPB3	AT2G15430	2	0	0	0
Reductase C	AT2G41680	3	0	0	81
RNA‐binding	AT3G15010	5	0	0	0
Unknown	AT5G42765	3	0	0	6
YAK1	AT5G35980	5	0	0	0

^a^ Spectra corresponding to the protein are shown. Two technical replicates gave similar results and were merged.

**Figure 6:**
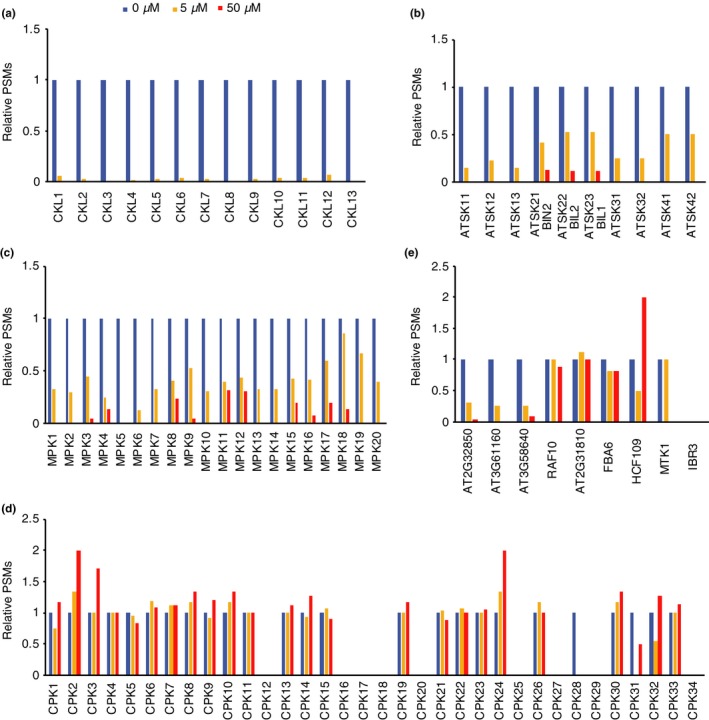
Relative spectra of potential PHA767491 target proteins in AMI‐329 bead binding. Relative peptide spectrum matches (PSMs) for CKL family (a), ATSK family (b), MPK family (c), CPK family (d), and the other proteins that are possible targets of PHA767491 (e). Spectra normalized to “0 µM” values are shown from AMI‐329 bead binding assays. Although we used only proteins with digested peptide spectra that were > “2” in the no‐competitor (0 µM) samples to ensure data integrity in Table 1 to find targets of AMI‐331, we used data include whose peptide spectra = 1 in 0 µM samples in Figure 6. Actual PSMs are shown in Figure S4

To examine other potential targets of AMI‐331, we analyzed the spectra for PHA767491 target proteins (Uehara et al., 2019). Spectra of ATSK (GSK3) family proteins were “2” to “31” in the 0 µM AMI‐331 sample (Figure S3). These spectra were 14 to 53% in the 5 µM sample, and 0%–13% in the 50 µM sample (Figure 6), suggesting that AMI‐331 has some binding affinity for ATSKs. Except for MPK5, spectra of the MPK family were present at a reliable range (2–52 PSMs) in the 0 µM sample (Figure S4). MPK spectra were 13%–85% in the 5 µM samples, and 0%–31% in the 50 µM samples, suggesting that AMI‐331 binds weakly to MPKs (Figure 6). Although CPK6 and CPK26 are possible targets of PHA767491 (Uehara et al., 2019), not all of the CPK family members were enriched in the 0 µM sample compared to the 5 or 50 µM samples, indicating that CPK is not a target of AMI‐331 (Figure 6). Three other protein kinases (AT2G32850, AT3G61160, and AT3G58640), that are candidates as targets of PHA767491, were also enriched in the 0 µM sample compared to 5 and 50 µM samples, suggesting that these proteins are also targets of AMI‐331 (Figure 6). These quantitative data suggest that although AMI‐331 binds to some PHA767491 targets, the specificity of AMI‐331 for CK1 family kinases was much greater than PHA767491.

### 3.6 Effect of AMI‐311 on expression of CK1 and ATSK downstream genes

Because the ATSK family was identified by the molecular probe assay as potential targets of AMI‐331, even though the affinities between AMI‐331 and ATSKs were not as high as between AMI‐331 and CK1, we used the ATSK inhibitor Bikinin to determine if AMI‐331 treatment affects the expression of genes downstream of ATSKs (De Rybel et al., 2009). Four‐day‐old Arabidopsis seedlings grown under constant light conditions were treated with Bikinin or AMI‐331 for 6 hr, and mRNA extracted from seedlings samples was analyzed by RT‐qPCR.

**Figure 7:**
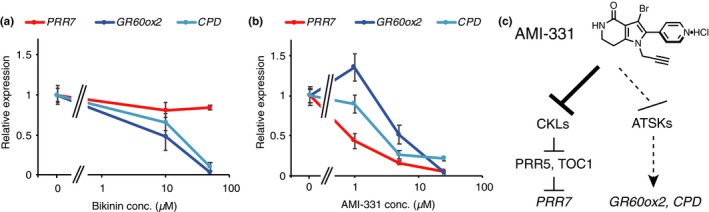
Transcript abundances of *PRR7*, *GR60ox2*, and *CPD* genes in plants treated with AMI‐331. (a) Plants were treated with DMSO, 10 µM or 50 µM of Bikinin for 6 hr, and gene expression at the transcript abundances was compared by qRT‐PCR. (b) Plants were treated with equal volumes of DMSO only, 1, 5, or 20 µM of AMI‐331 dissolved in DMSO. Error bars indicate *SEM* of three biological replicates. Note that the same DMSO samples were used for (a) and (b). (c) AMI‐331 activity for clock‐related gene *PRR7* and brassinosteroid‐related genes *GR60ox* and *CPD*. *PRR7* is regulated by CKLs, and *GR60ox2* and *CPD* are regulated by ATSKs

Treatment with 10 or 50 µM Bikinin reduced the transcript abundance of the ATSK downstream genes *GR60ox2* and *CPD* (Figure 7a) (De Rybel et al., 2009). Bikinin treatment at 10 and 50 µM did not affect transcript abundance of *PRR7*, a downstream gene of the CK1 family. This result suggests that Bikinin targets ATSK but not CK1.

Treatment with 1 µM AMI‐331 significantly decreased *PRR7* transcripts, but not *GR60ox2* or *CPD*. AMI‐331 at higher concentrations (5 and 25 µM) decreased *PRR7*, *GR60ox2*, and *CPD* transcript abundances (Figure 7b). The effect of 5 µM of AMI‐331 on *PRR7* was stronger than on *GR60ox2* and *CPD*. These results suggest that AMI‐331 targets CKLs most effectively, but also targets ATSKs in vivo (Figure 7c).

## 4 DISCUSSION

### 4.1 CK1 inhibitory activity of AMI‐331 in vivo

In this work, we present a new and potent inhibitor of CK1 family kinases, derived from the lead or seed molecule PHA767491. Generally, uptake by roots or through above‐ground epidermal tissues, solubility, transport in and out of cells, metabolic turnover, and inhibitory activity of targets can affect and restrict the activity of pharmacologically active molecules in plants. One reason for anticipating the strong in vivo inhibitory activity of AMI‐331 was its in vitro CK1 inhibitory activity. However, the IC_50_ of AMI‐331 on CKL4 kinase activity was only sevenfold less than PHA767491, but the period‐lengthening activity of AMI‐331 was 100 times greater than for PHA767491. In addition, the effective concentrations for increasing PRR5 and TOC1 expression by AMI‐331 were about 10–50 µM, or 10–50 times lower than required for PHA767491. These results suggest that factors such as cell membrane permeability, metabolic turnover, and the intracellular location of AMI‐331 all may contribute to the strong AMI‐331 activity in vivo.

### 4.2 High selectivity of AMI‐331 for CK1

A previous study suggested that PHA767491 targets the CKL family, resulting in clock dysregulation (Uehara et al., 2019). However, PHA767491 can also bind to ATSK, CPK, MPK, and other kinases, a confounding factor for interpreting the binding selectivity of PHA767491 for CKL or other proteins involved in clock period regulation (Uehara et al., 2019).

In this study, target identification using AMI‐329‐bound beads suggested that the most specific targets of AMI‐331 are in the CK1 family. Addition of 5 µM AMI‐331 mostly eliminated binding between AMI‐329 beads and CKL proteins (Figures 5 and 6). AMI‐331 binds to each CKL member (Table 1), as would be predicted from the similarity of amino acid sequences within the CKL kinase domains. Although AMI‐331 allows a greater ability to separate the CK1 family kinases from other kinases for clock activity, in the absence of finer levels of specificity, the dissection of individual CKL member functions by small molecules remains technically challenging. Generation of single and multiple CKL mutants through genome editing technology, perhaps in concert with small inhibitor molecules, holds promise for dissecting out individual CKL protein functions.

It has previously been suggested that PHA767491 acts as a competitor of ATP at the ATP‐binding pocket of target proteins (Uehara et al., 2019). In this study, we found that most AMI‐331 target proteins are CKLs that have ATP‐binding pockets (Table 1), and the structures of AMI‐331 and ATP have some similarity, suggesting that AMI‐331 acts as a competitor of ATP within the CK1 kinase family, but not in most other kinases. Because 5 µM AMI‐331 decreased binding between AMI‐329 beads and ATSK to between 50% and 20% of control samples without AMI‐331, higher concentrations of AMI‐331 may also bind to ATSK family kinases. In addition, gene expression assays showed that 1 µM of AMI‐331 affects expression of *PRR7*, which is regulated by CKLs, but not either *GR60ox2* or *CPD*, which are phosphorylated downstream of ATSKs. These results suggest that AMI‐331 preferentially targets the CKL family over the ATSKs. But it is also clear that AMI‐331 modulates ATSK activity in vivo because targets downstream of the ATSKs were changed by AMI‐331 treatment (Figure 7). MPKs are not so specifically bound by AMI‐331 (Figure 5). AT2g32850 (protein kinase) and MTK1, which were highly enriched by PHA767491‐bound beads, were not enriched by the AMI‐329 beads at all. These lines of evidence suggest that the selectivity of AMI‐331 is greater than PHA767491 toward the CKL family. Although AMI‐331 binds to ATSKs and modulates gene expression downstream of ATSKs (Figures 6 and 7), inhibition of ATSKs by Bikinin did not result in period lengthening (Uehara et al., 2019), suggesting that ATSKs are not involved in clock regulation. Collectively, the use of the novel, highly potent, and specific CK1 inhibitor AMI‐331 makes it possible to propose a new model in which inhibition of only the CKL family, but not ATSKs, MPKs, CPKs, or MTK1, is responsible for circadian period lengthening.

### 4.3 Possible uses for AMI‐331 in plant biology

Most plants have multiple CKLs, and there are at least 13 members of the CKL family in Arabidopsis. The presence of multiple CKL‐encoding genes in the plant genome complicates the identification of the physiological functions controlled by CKLs. We have shown that PHA767491 is a CK1 inhibitor in plants, but the effective concentration of PHA767491 is more than 40 µM (Uehara et al., 2019). By contrast, AMI‐331 effectively lengthened the clock period at concentrations below 1 µM. In addition, 10–50 µM AMI‐331 increased PRR5 and TOC1 accumulation, and 1 µM AMI‐331 decreased *PRR7* expression. Lower effective concentrations of pharmacologically active molecules are beneficial because it minimizes off‐target effects. The high specificity and low effective concentrations of AMI‐331 in vivo should enable researchers who work with evolutionarily divergent eukaryotic model systems to judge whether or not the CK1 family is involved any given physiological processes. Additional opportunities may arise in plants or other organisms about which little genetic information is known, such as minor crop species, or in plants that are recalcitrant to genetic manipulation.

Because the circadian clock controls many physiological processes, such as stress responses and flowering time regulation, clock modulators potentially become agricultural regents (Uehara et al., 2019). However, PHA767491, the parent molecule of AMI‐331, reportedly inhibits mammalian CK1 and CDC7 proteins that are involved in essential roles in development and DNA replication, respectively (Montagnoli et al., 2008; Uehara et al., 2019). Thus, using AMI‐331 itself for agricultural purposes does not seem to hold much promise, unless it is shown that AMI‐331 does not modulate CK1 and CDC7, or other enzymes of non‐plant organisms. It is worthy to note that higher concentrations of AMI‐331 may also modulate physiological processes through CKL‐independent pathways (i.e., off‐target effects). Consequently, the possibility of additional unknown or unexpected effects of AMI‐331 cannot be excluded if AMI‐331 binds to non‐protein molecules.

## CONFLICT OF INTEREST

The authors declare no conflict of interest associated with the work described in this manuscript.

## AUTHOR CONTRIBUTIONS

JY and NN designed the research plan; ANS and JY synthesized small molecules; HM, AO, and NN performed the experiments; KK performed proteomics analysis; HM, KK, AO, TK, and NN analyzed data; and JY and NN wrote the paper.
