# Supplementary material for: Structure–function study of a novel inhibitor of the casein kinase 1 family in Arabidopsis thaliana
Source: Plant Direct. 2019 Sep 17;3(9):e00172. doi: 10.1002/pld3.172 (PMC6747015; doi:10.1002/pld3.172)
Supplement: Supplementary file 1 [file PLD3-3-e00172-s001.pdf]

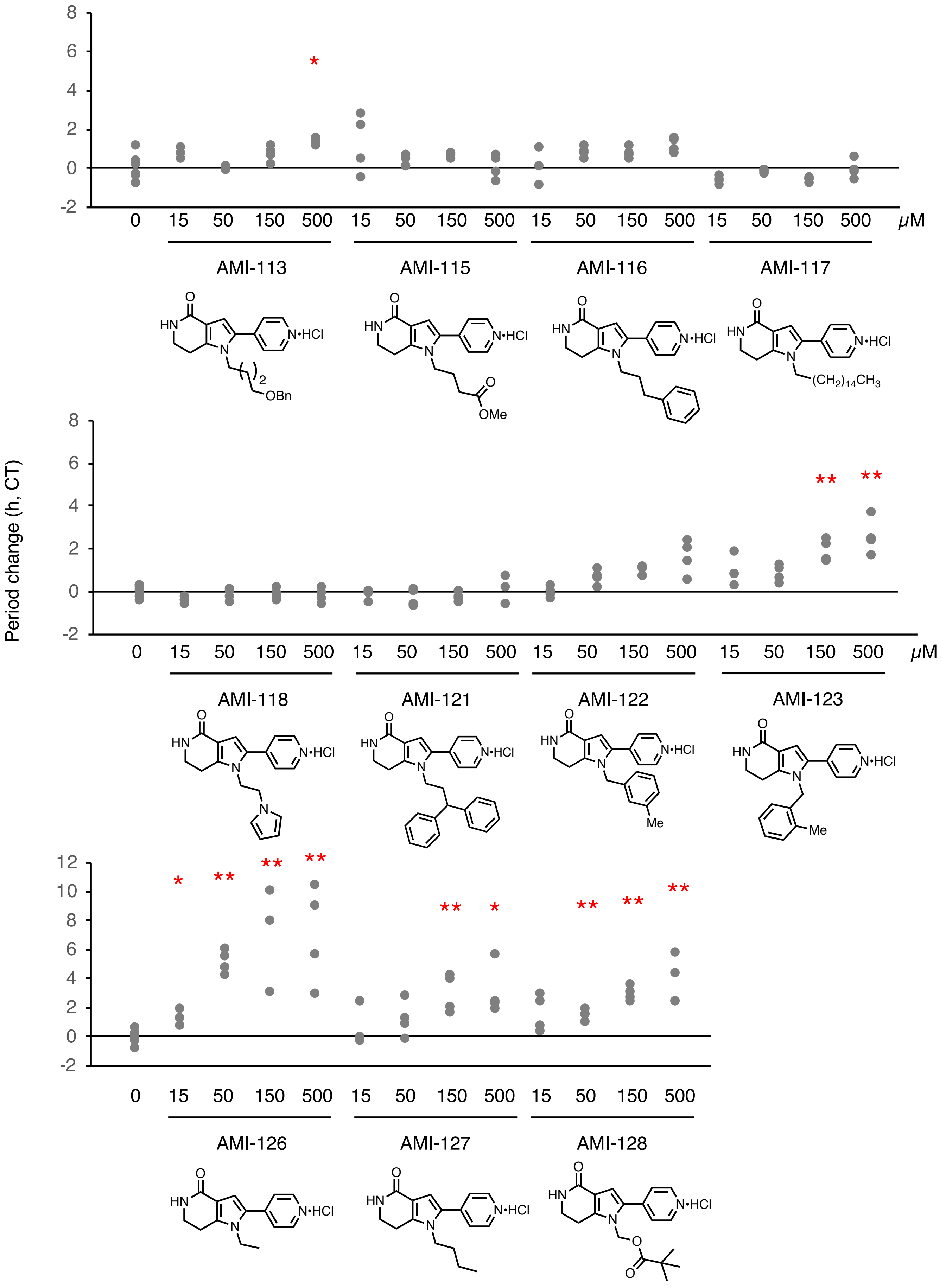

Sup. Figure 1 (1)

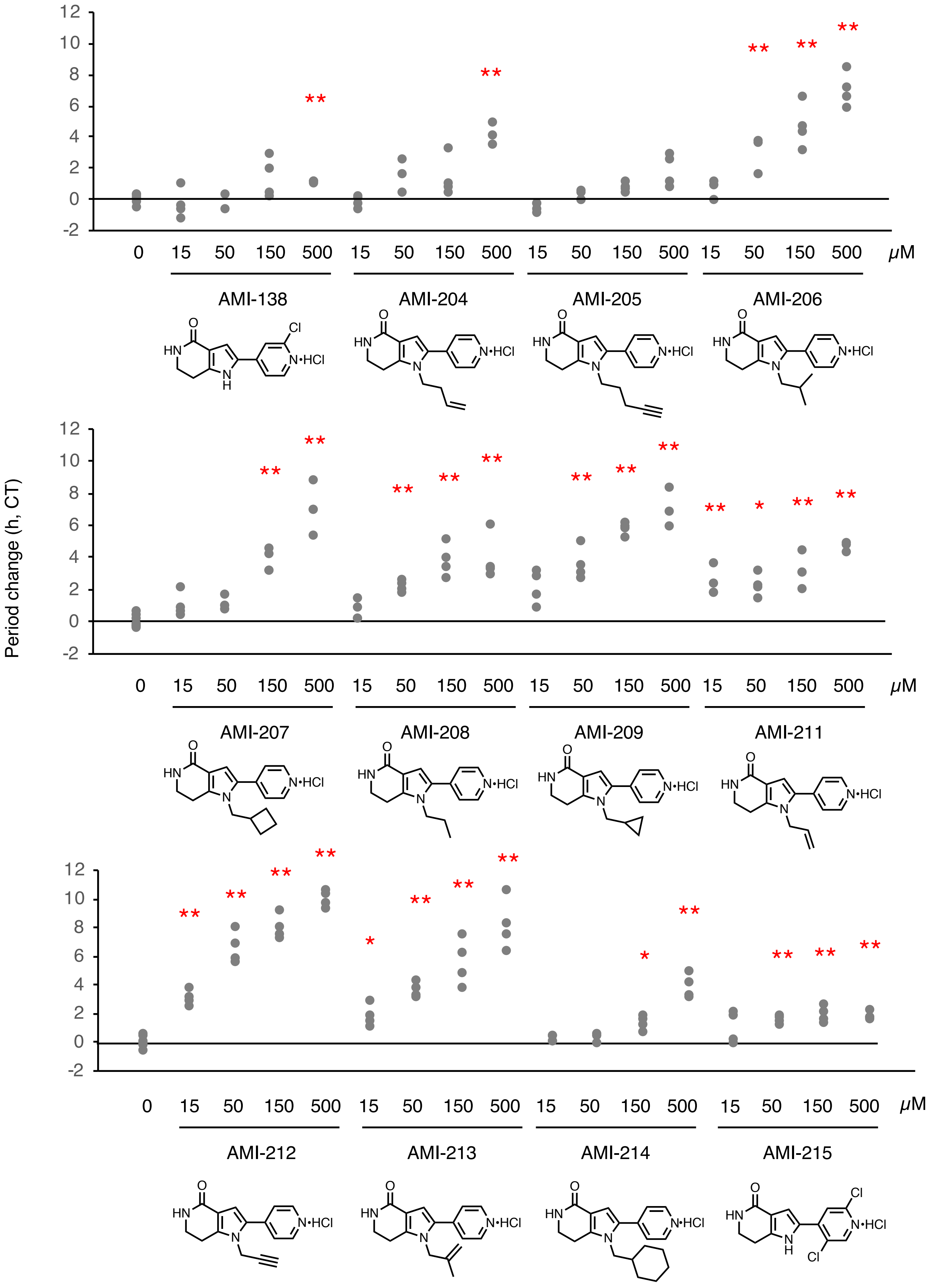

Sup. Figure 1 (2)

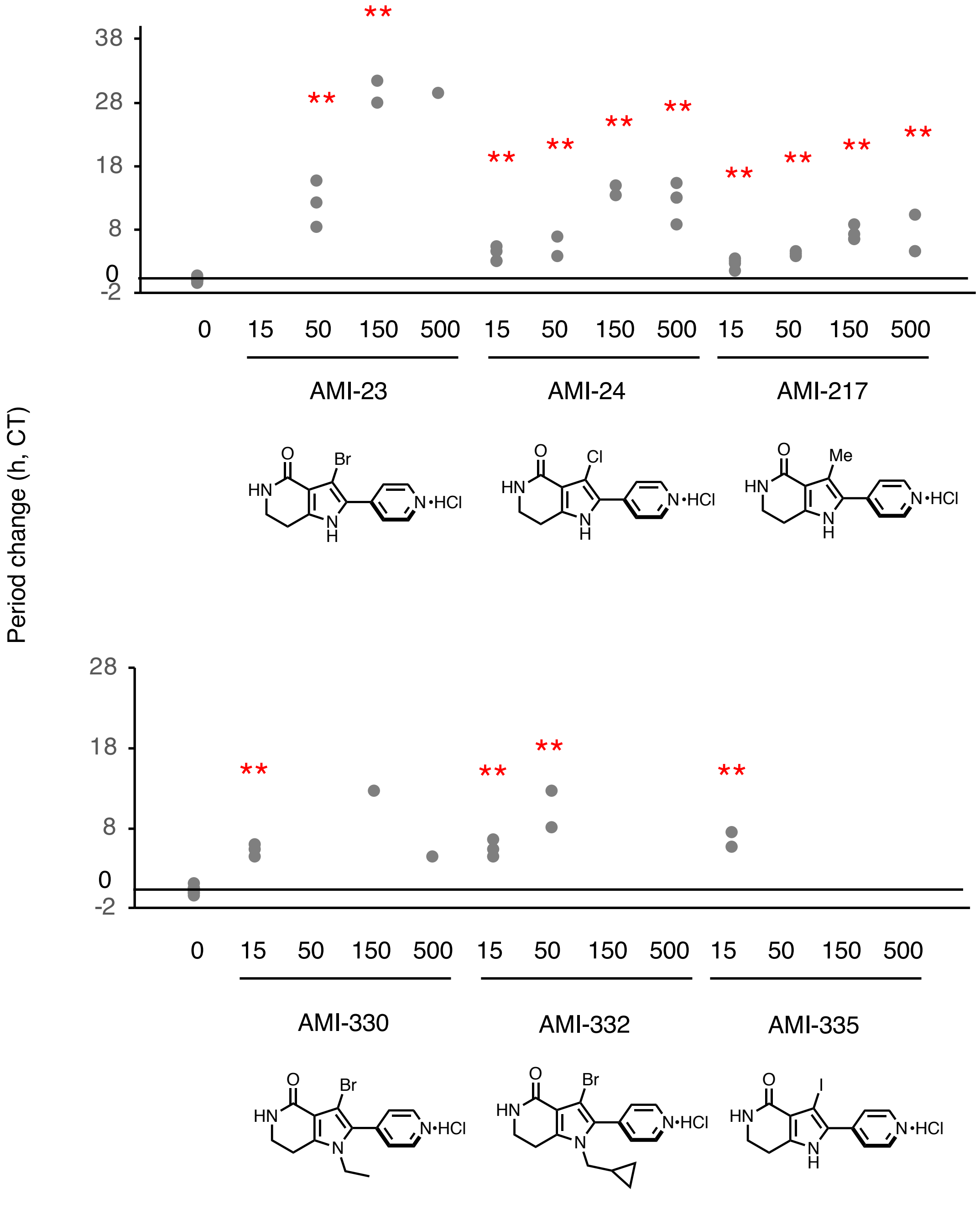

Supplemental Figure 1. Period-lengthening activities of PHA767491-derivatives (AMI molecules). Each dot represents the difference between CT-corrected period length and the DMSO control. Asterisks and double asterisks indicate significant period changes compared to control samples (Bonferroni correction $p < 0.05$ and 0.01, respectively).

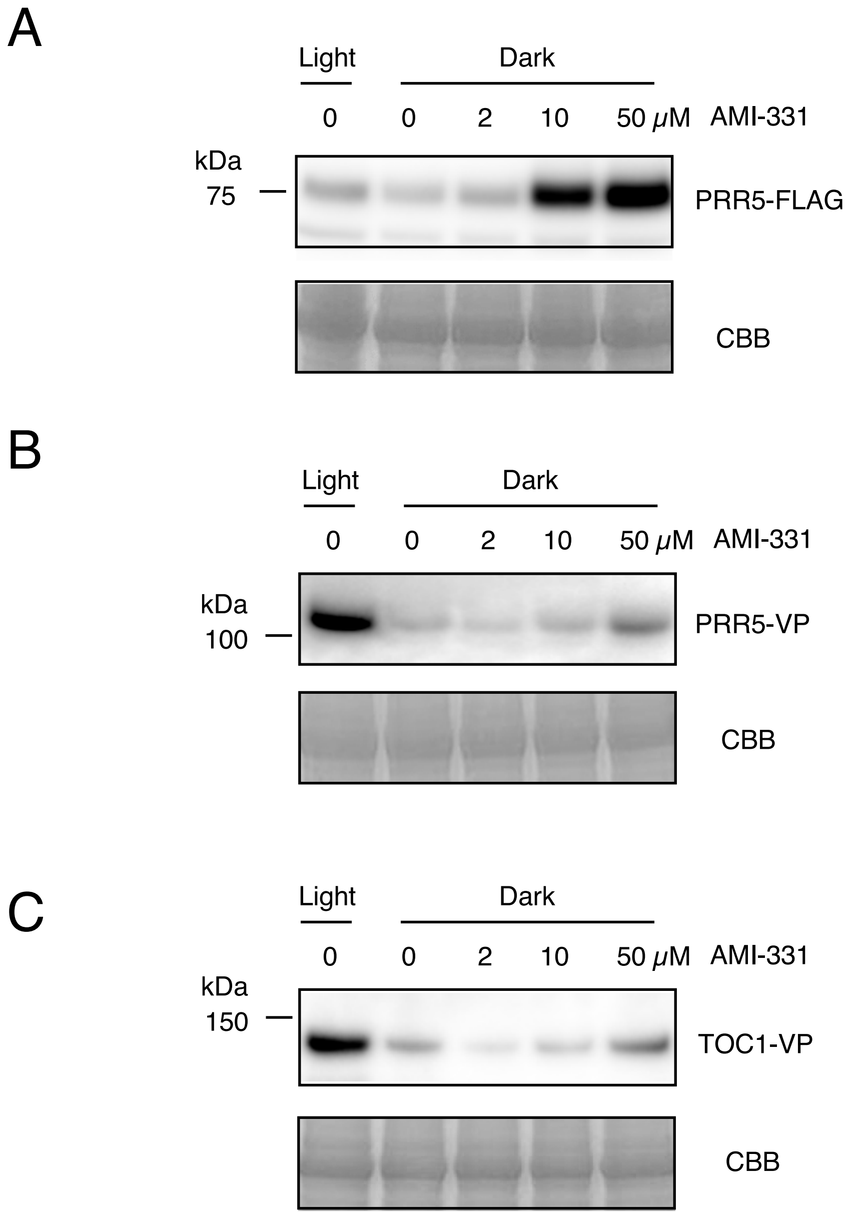

Supplemental Figure 2. Confirmatory assay for data shown in Figure 3 (PRR5 and TOC1 protein accumulation in plants treated with AMI-331). Accumulation of PRR5-FLAG **(A)**, PRR5-VP **(B)**, and TOC1-VP **(C)** proteins in transgenic plants treated with AMI-331 (upper panels). CBB acts as a loading and normalization control.

Sup. Figure 2

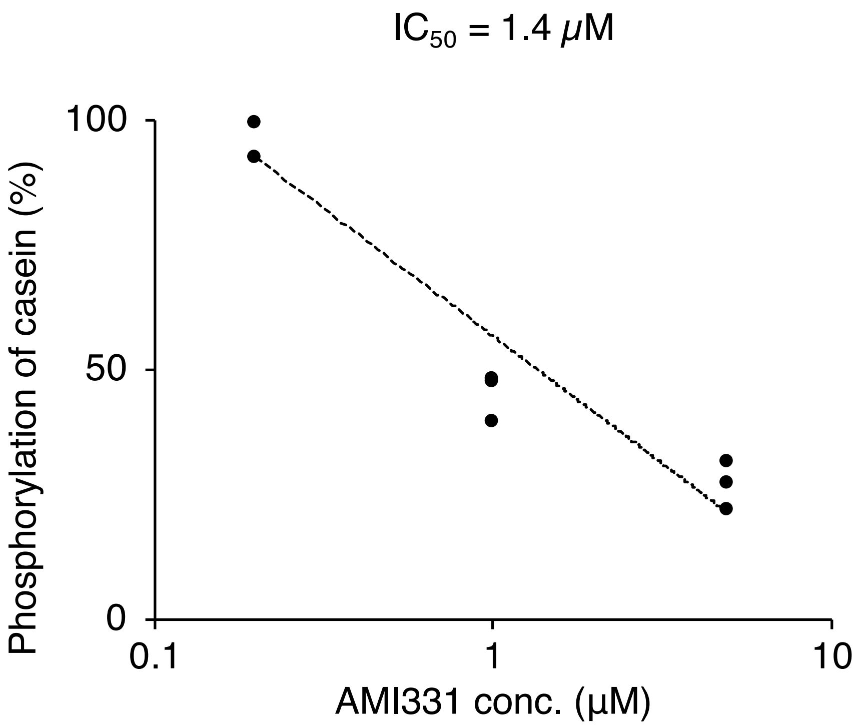

Supplemental Figure 3. In vitro CKL1 kinase activity with AMI-331.

Sup. Figure 3

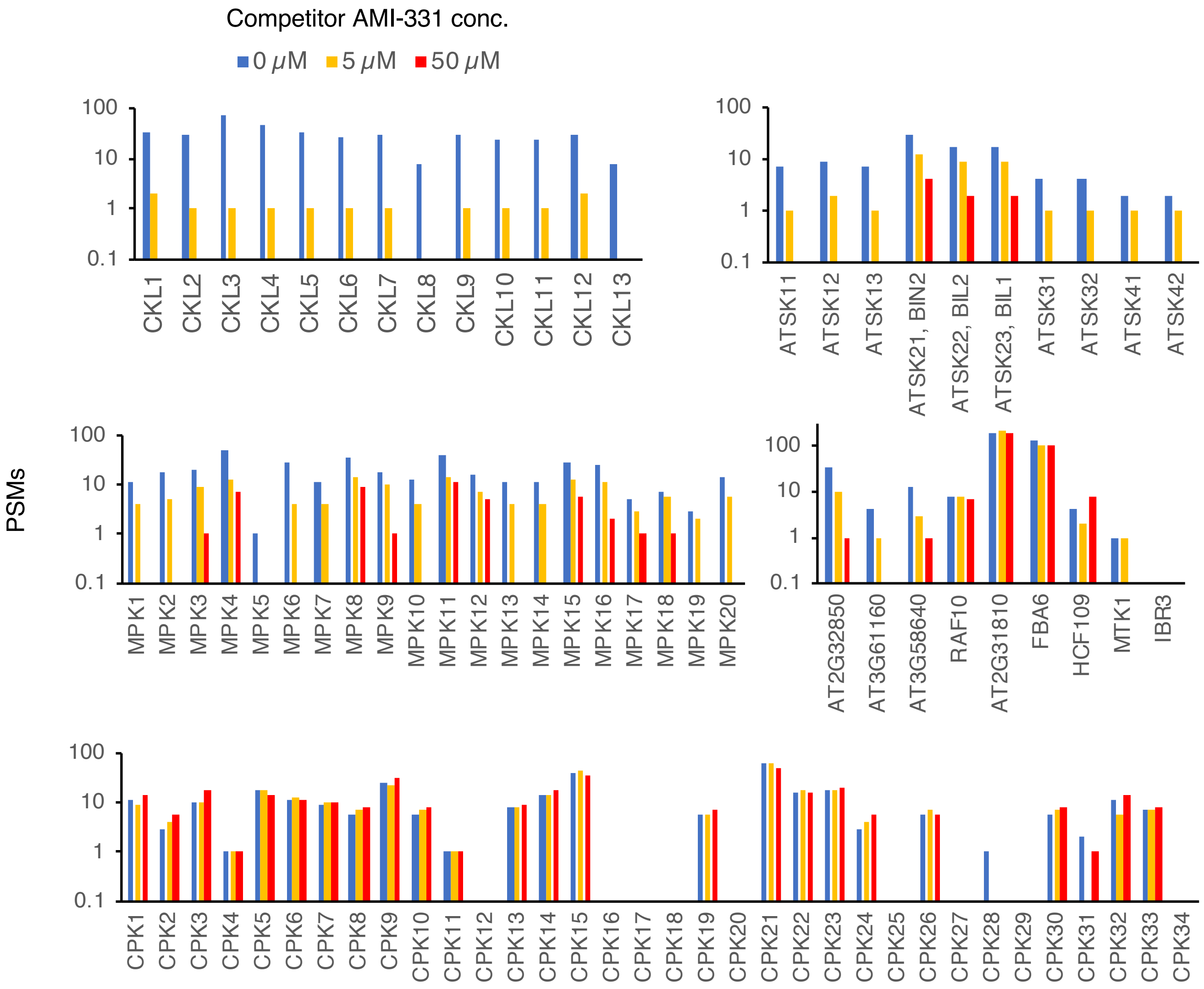

Supplemental Figure 4. Spectra of potential PHA767491 target proteins in AMI-329-bead binding assays.

Sup. Figure 4
